# Genetic Loci Involved in Antibody Response to *Mycobacterium avium ssp. paratuberculosis* in Cattle

**DOI:** 10.1371/journal.pone.0011117

**Published:** 2010-06-15

**Authors:** Giulietta Minozzi, Laura Buggiotti, Alessandra Stella, Francesco Strozzi, Mario Luini, John L. Williams

**Affiliations:** 1 Parco Tecnologico Padano, Polo Universitario, Lodi, Italy; 2 Istituto Sperimentale Italiano Lazzaro Spallanzani, Lodi, Italy; 3 Istituto di Biologia e Biotecnologia Agraria, Consiglio Nazionale delle Ricerche, Lodi, Italy; 4 Istituto Zooprofilattico Sperimentale della Lombardia e dell'Emilia Romagna, Lodi, Italy; Smithsonian Institution National Zoological Park, United States of America

## Abstract

**Background:**

*Mycobacterium avium subsp. paratuberculosis (*MAP*)* causes chronic enteritis in a wide range of animal species. In cattle, MAP causes a chronic disease called Johne's disease, or paratuberculosis, that is not treatable and the efficacy of vaccine control is controversial. The clinical phase of the disease is characterised by diarrhoea, weight loss, drop in milk production and eventually death. Susceptibility to MAP infection is heritable with heritability estimates ranging from 0.06 to 0.10. There have been several studies over the last few years that have identified genetic loci putatively associated with MAP susceptibility, however, with the availability of genome-wide high density SNP maker panels it is now possible to carry out association studies that have higher precision.

**Methodology/Principal Findings:**

The objective of the current study was to localize genes having an impact on Johne's disease susceptibility using the latest bovine genome information and a high density SNP panel (Illumina BovineSNP50 BeadChip) to perform a case/control, genome-wide association analysis. Samples from MAP case and negative controls were selected from field samples collected in 2007 and 2008 in the province of Lombardy, Italy. Cases were defined as animals serologically positive for MAP by ELISA. In total 966 samples were genotyped: 483 MAP ELISA positive and 483 ELISA negative. Samples were selected randomly among those collected from 119 farms which had at least one positive animal.

**Conclusion/Significance:**

The analysis of the genotype data identified several chromosomal regions associated with disease status: a region on chromosome 12 with high significance (P<5×10^−6^), while regions on chromosome 9, 11, and 12 had moderate significance (P<5×10^−5^). These results provide evidence for genetic loci involved in the humoral response to MAP. Knowledge of genetic variations related to susceptibility will facilitate the incorporation of this information into breeding programmes for the improvement of health status.

## Introduction

*Mycobacterium avium* subspecies *paratuberculosis* (MAP) causes paratuberculosis or Johne's disease in cattle, a chronic granulomatous gastroenteritis [1], [2]. Johne's disease occurs worldwide and is primarily a disease of ruminants, including cattle, sheep, goats, and farmed deer. However, the disease has a wide host range and has been reported to occur in non-ruminants species, such as wild rabbits [3] and their predators, foxes and stoats [4], and in primates such as mandrills and macaques [5], [6].

MAP is responsible for huge economic losses, particularly in dairy cattle herds [7]. Moreover, several studies have suggested a link between MAP and Crohn's disease in man [8]–[10]. However, the evidence for a link between Johne's and Crohn's diseases remains controversial and the causal role of MAP has not been proven [11]–[13].

In cattle, the disease starts with the slow development of intestinal lesions in infected animals, a proportion of these animals become clinically ill after two to six years [14]. Clinical signs of infection include progressive weight loss, intractable diarrhoea, decreased milk production and ultimately death [15]. However, in cattle, Johne's disease is not treatable and vaccine efficacy it is still controversial. The prevalence of MAP in farmed animals in Europe is approximately 20% [16].

The main route of transmission of MAP is the faecal-oral route [17]; however, it can also be transmitted in the semen of bulls, in milk to the newborn calf, and *in utero* across the placenta [2]. In addition it has also been suggested that MAP can exist within the tissues of animals for years without causing clinical disease [18], [19]. Although the mechanisms that affect the balance between acquired resistance and progression to clinical disease are unknown, they may involve maturation of the immune system in terms of the various T-cell subsets and the specific tissue distribution of immune cells. In the early stages of the infection, MAP infects macrophages in lymphoid tissue in the ileum, where it inhibits phagosome maturation and induces the recruitment of inflammatory cells, resulting in granulomatous enteritis. Cattle typically become infected with MAP as calves; however, clinical signs of infection do not usually appear before two years of age, and are most commonly seen after the second or third lactation. Infected cattle may spread MAP to other animals in the herd through faecal contamination of the environment, prior to the appearance of clinical signs [20]. Current Johne's diagnostic tests have low sensitivity for detecting the infection in pre-clinical animals (0.45–0.5) [21], thus testing for MAP may not identify all infected animals. The sub-clinical stage of MAP infection is characterized by loss of pro-inflammatory Th1 response and an increased antibody-mediated Th2 response, however, the mechanism by which MAP interacts with the bovine immune system and suppresses Th1 response remains unclear [22].

Susceptibility to MAP infection has been found to be heritable [23]–[26] with heritability estimates ranging from 0.06 to 0.102, depending the definition of infection, the statistical model used and the population studied.

Several studies have addressed the identification of genetic loci associated with MAP susceptibility by testing candidate genes, by genome-wide linkage or association studies. Polymorphisms in functional candidate genes, *SLC11A1* [27], *TLR1*, *2* and *4*, [28] and *CARD15* [29] have been associated with susceptibility with MAP infection in cattle. *CARD15* has also been associated with increased the risk of Crohn's disease in humans [30], [31]. Genome wide linkage analysis provided evidence for a QTL for MAP susceptibility on *Bos taurus* chromosome 20 [32] and recently, a genome wide association study using a high density single nucleotide polymorphism (SNP) panel (the Illumina BovineSNP50 BeadChip) identified regions on chromosomes 3 and 9 that are highly significantly associated with the presence of MAP in tissue and faeces [33]. However, neither of these publications present evidence for strong functional candidate genes associated with Johne's disease in these chromosomal regions.

The current study presents evidence for loci associated with MAP susceptibility that were identified using a high density SNP panel (the Illumina BovineSNP50BeadChip whole genome SNP assay) in a case-control study with a sample size of about 900 Holstein cattle using the presence of antibody against the bacterium as the definition of susceptibility.

## Results

### 1. Genotype quality assurance and internal population structure analysis

Following quality control checks, 846 of the 54,001markers were excluded because of low (<95%) call rate, 6511 markers were excluded because of low minor allele frequency (MAF) <0.002 and 294 markers were excluded because they were out of Hardy-Weinberg equilibrium in controls at a false discovery rate (FDR <0.2). With respect to the samples: 26 were removed because of low call rate (<0.95) and 1 sample was eliminated because of high autosomal heterozygosity (FDR <1%). The mean heterozygosity of the sample was 0.33±0.01, while the sample removed had heterozygosity higher than 0.53, indicating possible sample contamination. A further 14 samples were removed due to high IBS (Identity By State). Mean IBS was 0.73±0.01, based on 2000 autosomal markers, while the samples removed showed IBS >0.95. No outliers were identified by Classical Multi Dimension Scaling (MDS), consequently the final data set that passed the quality controls and was used in the association analysis contained 46350 Genome wide SNPs and 925 samples.

### 2. Genome Wide Association Analysis

**Figure 1:**
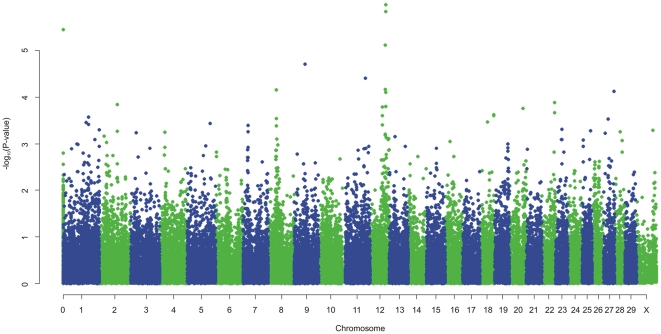
Manhattan plot displaying the results (−log10 of *p*-value) of the Genome-wide scan using the GRAMMAR-GC method with respect to their genomic position.

**Figure 2:**
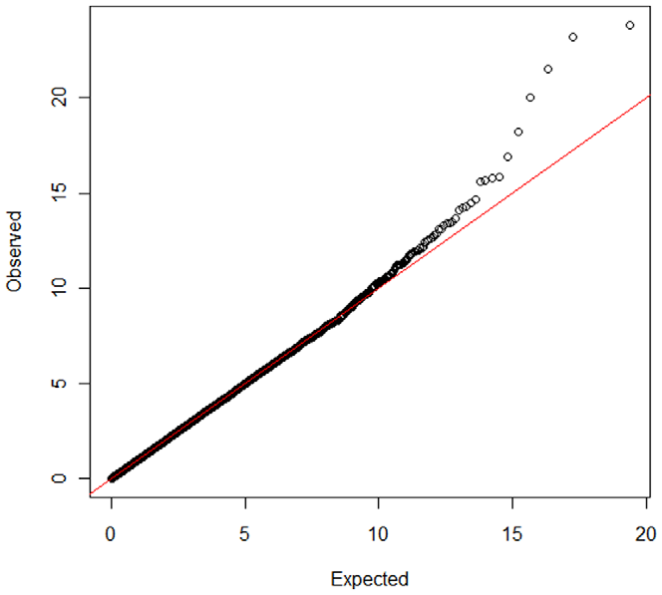
Q-Q plot of (GRAMMAR-CG) p-values/against expected p-values.

Genome Wide Analysis identified SNPs with significant association with MAP antibody response on chromosomes 12, 8, 9, 11, and 27. Genome-wide Manhattan plots displaying the GWA results with respect to their genomic position, are shown in Figure 1 and details are given in Table 1. All the markers with significant associations had high call rates, ranging between 0.97 and 0.99, and MAF ranging between 0.11 and 0.42 (Table 1). Evidence of population substructure was estimated by the genomic inflation factor λ = 1.15 for a basic chi-square statistics test, and was completely corrected by the GRAMMAR-CG methodology that yielded λ = 1. The Q-Q plot of the resulting analysis is shown in Figure 2.

**Table 1:** List of SNP associated with positive ELISA test to MAP infection indentified by Genome Wide Association study in Holstein cattle.

SNP name	BTA	Position (bp)	N	effB Q.2	P1df	Pc1df	Call Rate	Q.2	P.11	P.12	P.22	Pexact H-W test	Coding Minor/major allele
ARS-BFGL-NGS-57278	12	69663832	903	0.157	2.29e-06	1.04e-06	0.97	0.11	722	167	14	0.22	G/A
BTA-95991-no-rs	12	69599639	925	0.154	3.14e-06	1.44e-06	1	0.11	743	167	15	0.12	G/A
BTB-01626215	0	0	909	0.168	7.28e-06	3.55e-06	0.98	0.10	732	177	0	0.0001	G/A
ARS-BFGL-NGS-101584	12	68553182	925	0.134	1.50e-05	7.70e-06	1	0.12	709	198	18	0.37	G/A
ARS-BFGL-NGS-8531	9	46362363	925	0.097	3.59e-05	1.94e-05	1	0.27	496	362	67	0.93	A/G
ARS-BFGL-NGS-17731	11	89695127	923	−0.082	6.95e-05	3.93e-05	0.99	0.42	310	445	168	0.73	A/G
ARS-BFGL-NGS-105846	12	67342543	925	0.113	1.17e-04	6.83e-05	1	0.15	659	247	19	0.53	A/G
BTB-02056135	8	37257076	925	−0.084	1.19e-04	7.02e-05	1	0.37	356	446	123	0.40	A/C
ARS-BFGL-NGS-37647	27	45253563	925	−0.086	1.19e-04	7.55e-05	1	0.11	321	484	120	0.003	A/G
BTB-01470661	12	69808111	900	0.135	1.32e-04	7.80e-05	0.97	0.11	696	203	1	4.75e-05	C/A

SNP name: snp name as in the bovine 50K SNP Chip data.

BTA: Bos Taurus Chromosome.

effB Q.2.: effect of the minor allele (B allele).

P1df: raw p-values before adjustment for Genomic Control.

Pc1df: p-values adjusted for Genomic Control.

Q.2.: frequency of the minor allele allele (B allele).

Pexact H-W test =  exact p-value for the test of Hardy-Weinberg estimated in cases and controls together.

**Figure 3:**
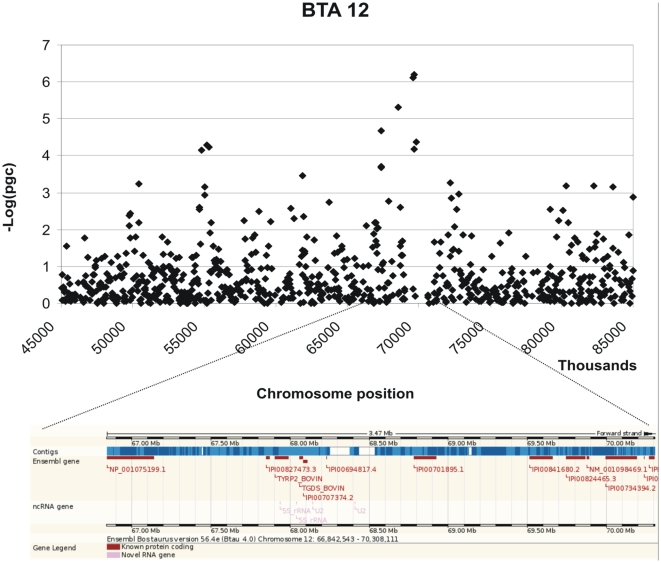
Scatter plots of the chromosomal region in BTA 12 associated with MAP and its corresponding genomic regions (taken from ENSEMBL).

Three moderately significant SNPs were identified on chromosome 12 at positions 69663832, 69599639 and 68553182 with p-values of 1.04 e-06, 1.44 e-06 and 1.50 e-05 and explained 0.48% 0.46% and 0.38% of the phenotypic variance respectively (Table 1). In addition 2 further SNPs on chromosome 12 at positions 67342543 and 69808111 showed p-values close to significance 6.84 e-05 and 7.80 e-05. These SNPs also had high call rates. The peak defining the region identified by the SNPs on chromosome 12 is bell shaped indicating a non random association (Figure 3). A list of the genes located within 1Mb from the significant SNP identified is provided in Table 2 and their potential functions are described in Table S1. A graphical representation of the chromosomal regions associated with MAP is shown in Figure 3–6. On chromosome 12 (BTA12) two of the significant SNPs are located in a coding region of the genes IPI00841680.2 and IPI00824465.3 respectively; both of these SNPs fall in the intronic region of the gene.

**Figure 4 pone-0011117-g004:**
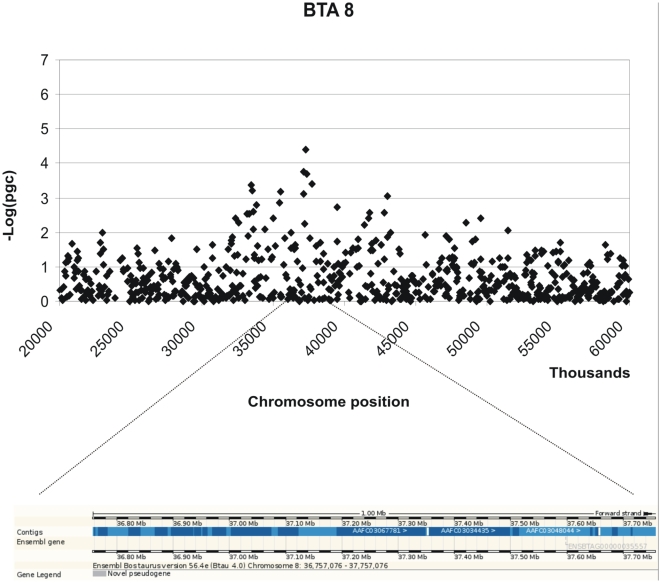
Scatter plots of the chromosomal region in BTA 8 associated with MAP and its corresponding genomic regions (taken from ENSEMBL).

**Figure 5 pone-0011117-g005:**
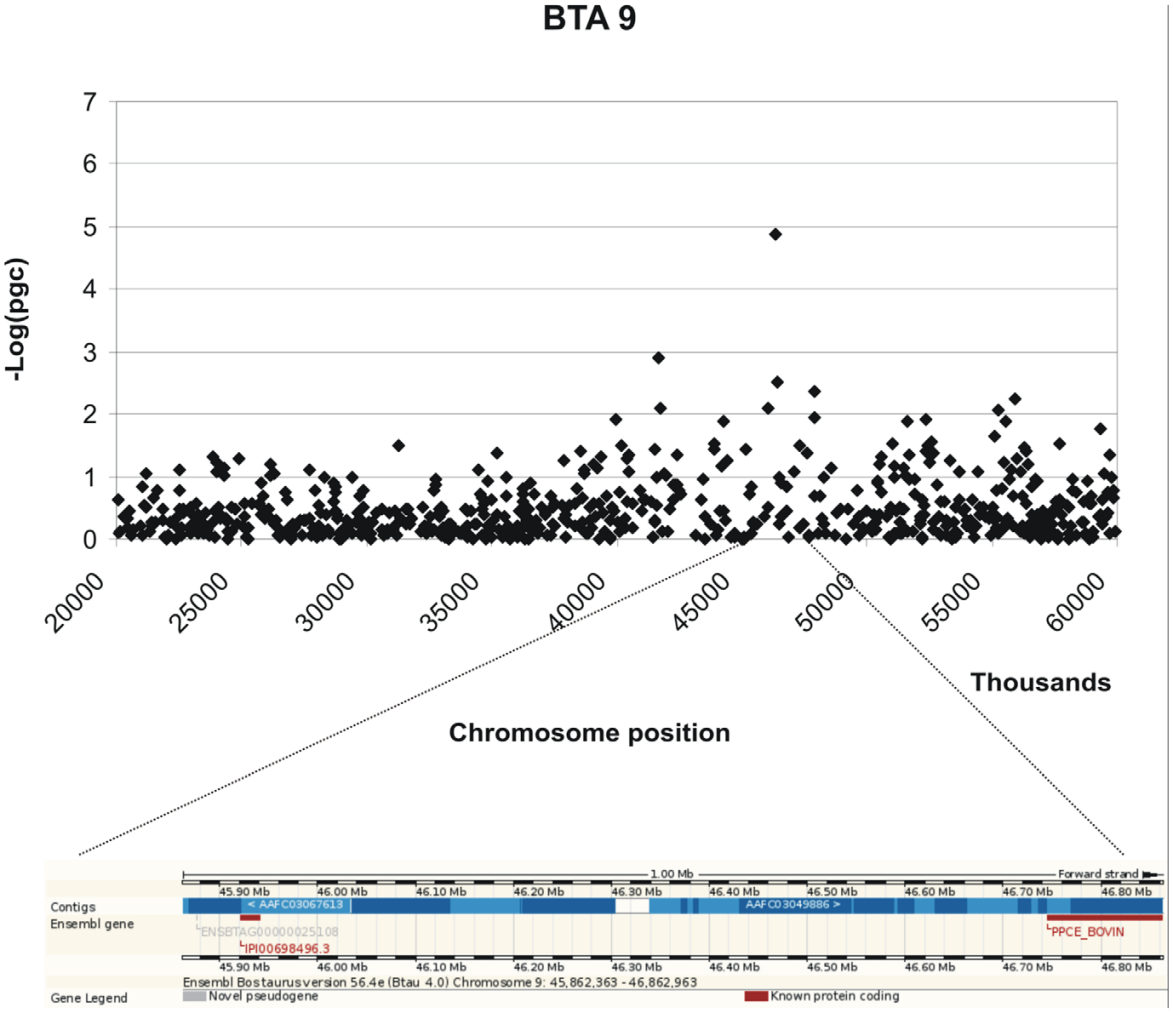
Scatter plots of the chromosomal region in BTA 9 associated with MAP and its corresponding genomic regions (taken from ENSEMBL).

**Figure 6:**
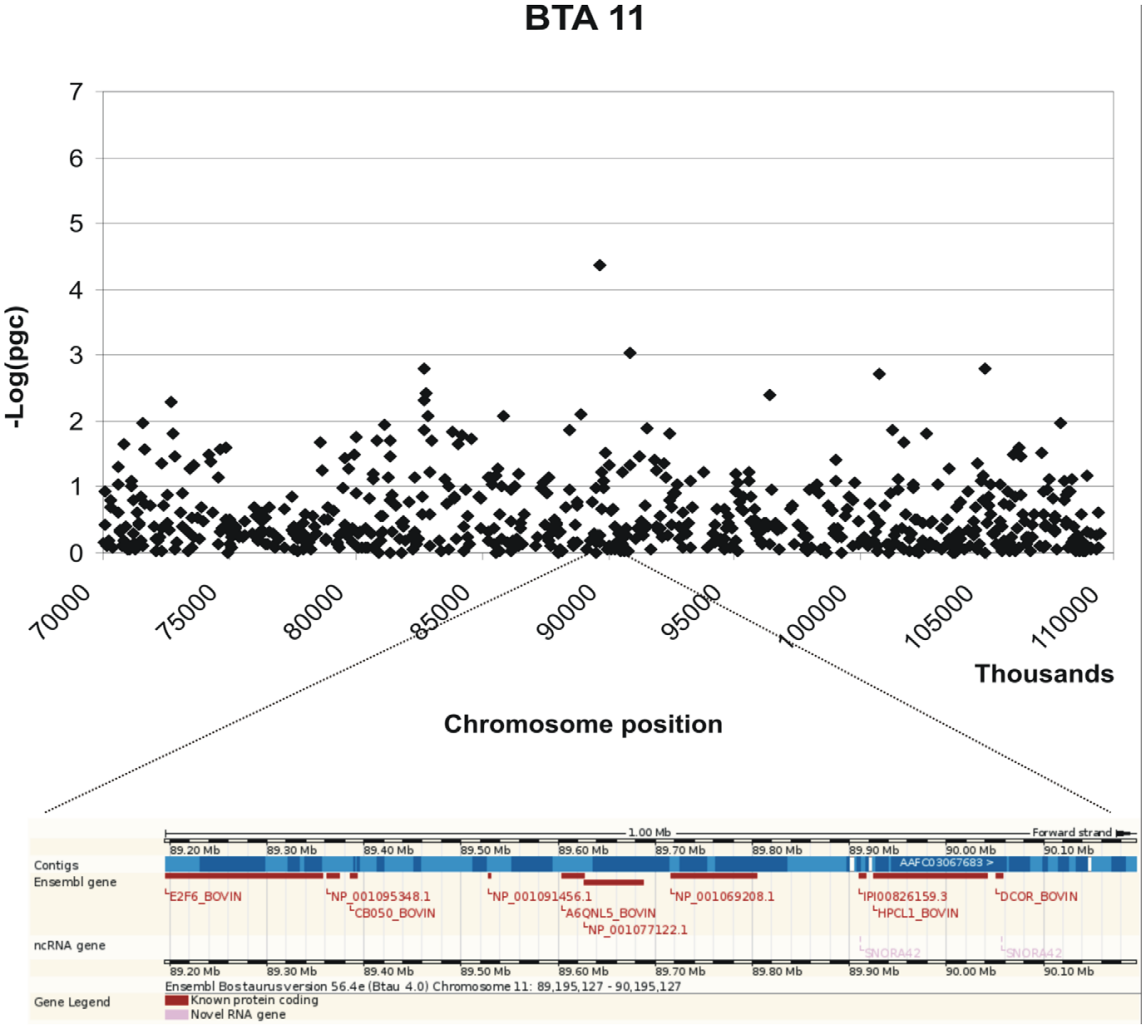
Scatter plots of the chromosomal region in BTA 11 associated with MAP and its corresponding genomic regions (taken from ENSEMBL).

**Table 2:** Genomic regions associated with MAP.

SNP	BTA	Position	P-value	RefSeq gene (1Mb)
ARS-BFGL-NGS-57278	12	69663832	1.04e-06	*GPC6, TYRP2, TGDS, GPR180, SOX21, ABCC4*
BTA-95991-no-rs	12	69599639	1.44e-06	
ARS-BFGL-NGS-101584	12	68553182	7.70e-06	
ARS-BFGL-NGS-105846	12	67342543	6.83e-05	
BTB-01470661	12	69808111	7.80e-05	
ARS-BFGL-NGS-17731	11	89695127	3.93e-05	*E2F6, PQLC3, C2o50, KCNF1, PDIA6, ATP6V1C2, NOL10, ODC1, HPCAL1*
ARS-BFGL-NGS-8531	9	46362363	1.94e-05	*PRDM1, PREP*
BTB-02056135	8	37257076	7.02e-05	None
ARS-BFGL-NGS-37647	27	45253563	7.55e-05	None
BTB-01626215	0	0	3.55e-06	None

Two further moderately significant SNPs were identified on chromosomes 9 and 11 (p-value of 1.94 e-05 and 3.93 e-05) at positions 46362363 and 89695126 explaining 0.34%, 0.33% and of the variance respectively. A further SNP with high significance (3.55 e-06) is currently not positioned on the bovine genome sequence. In addition, 2 SNPs had p-values close to significance: one on chromosome 8 at position 37257076 and the second on chromosome 27 at position 45253563, with p-values of 7.02 and 7.55 e-05 respectively (Table 1).

### 3. Confirmation in smaller cohort

The six SNPs with highest p-values were then tested using the same test statistics and by simple regression on a smaller cohort of 277 cases and MAP negative control Holstein animals drawn from the same population used for the main GWA and chosen using the same criteria as the main cohort. Case-control samples were randomly chosen to be herd, test day, sex matched, and to reduce the possibility of animals being related no more than 6 animals were selected from the same herd. No population substructure was identified (λ = 1). Following correction for multiple testing (n = 6), five of the six SNPs tested showed significant association with MAP infection, even in this the smaller cohort (Table 3). These SNPs were the two most significant SNP on BTA 12, the SNP on 9 and 11 and the un-unmapped SNP identified in the initial study. Even with the smaller sample size, the SNP on chromosome 11, at position 89695126, showed a p-value of 8.06 e-05 before correction for multiple testing, which was as high as the initial p-value of 6.95e-05 obtained using a larger number of animals. Following correction for multiple testing, the p-value reduced to 4.80e-04.

**Table 3:** Top 10 significant SNPs tested in the smaller (n = 277) cohort and corrected for multiple testing (n = 6).

SNP name	BTA	Position (bp)	N	P-value	Coding	p-value after muntiple correction (n = 6)
ARS-BFGL-NGS-57278	12	69663832	264	0.001**	G/A	0.006**
BTA-95991-no-rs	12	69599639	269	0.001**	G/A	0.006**
BTB-01626215	0	0	264	0.0002***	G/A	0.001**
ARS-BFGL-NGS-101584	12	68553182	269	0.016*	G/A	0.36
ARS-BFGL-NGS-8531	9	46362363	269	0.008**	A/G	0.048*
ARS-BFGL-NGS-17731	11	89695127	169	8.06e-05***	A/G	4.80e-4***

*P*≤0.05.

**P≤0.01.

***P≤0.001.

## Discussion

Considerable work has been carried out to address the genetic control of susceptibility and resistance to mycobacterial infections, including bovine paratuberculosis [23]–[25], [28], [29], [32]–[37]. Different approaches have been used to localise and identify the genes involved in susceptibility to MAP, including the testing of candidate genes [27] and QTL mapping using relatively low density markers [32]. More recently, following the publication of the bovine genome sequence, a genome wide association study (GWA) using higher density SNP panels has provided evidence of a genetic component in Jones's disease susceptibility [33].

In contrast to human association analysis where, in general, either small well characterised families, or unrelated individuals are used, the design of genome wide scans in livestock, based on field samples with unknown pedigree information, poses a series of different challenges. There is a high level of relatedness in cattle populations, especially for dairy cattle, where the effective population size and number of sires used for artificial insemination in the recent years is very small [38]. The hidden presence of closely related animals in the sample set can cause a complex population structure and an *a priori* unbalanced distribution of allele frequencies between cases and controls that is likely to inflate the rate of false positive associations between the trait and the markers, and could hide the true associations. Robust methodology has been developed to account for genetic background, based on a polygenic model and which is implemented in the GRAMMAR-CG approach used in the analysis presented here. This approach was able to disentangle the cryptic relatedness in the population by modelling the polygenic relatedness between pairs of samples [39].

In the present work a genome wide scan using a relatively high density of markers identified moderately significant associations between SNPs and MAP serology on chromosomes 9, 11 and 12. Several SNP markers on chromosome 12 were found to be significantly associated with MAP within a region of 1 Mb and formed a distinct peak together with the other SNPs that on their own fail to reach the threshold for significance (see Figure 1). Two of the significant SNPs are located in coding regions of two genes that code for the same protein, the ATP-binding cassette, sub-family C (*CFTR/MRP*), member 4 protein (*ABCC4*) which is a multi-drug resistance associated protein [40]. The possible role of this protein in response to Johne's disease is not known. A study that used DNA pools created from MAP affected and non affected animals from two sire families, compared allele frequencies for a panel of microsatellite markers between the pools and identified a marker significantly associated to MAP status on BTA12, located at position 67356645 [32]. This is within 2 Mb from the most significant SNP reported in the present study, and very close to a second SNP with a less significant association (p = 6.84 e-05) at position 67342543. The same QTL study also identified a region on BTA 20 associated with Johne's disease [32], however in the present study there was no evidence for an association with MAP status on this chromosome.

In the present study a SNP was identified on chromosome 9 at position 46362363 which was significantly associated with serologically defined MAP susceptibility. In another study the neighbouring SNP at position 46423922 was associated with MAP susceptibility defined by presence of bacteria in tissue of the ileum in Holstein cattle [33]. One interpretation of these findings may be that the genetic locus identified on chromosome 9 in these two studies is generally involved in the process of MAP infection and control of MAP replication, while the locus on chromosome 12 identified here, that was not identified in the study of Settles et al. [33], is more specifically involved in antibody response to this bacterium. This may indicate two or more distinct mechanisms of resistance that involve different metabolic pathways or different QTL segregating in different populations.

Two genes are located within 1 Mb, of the significant SNP on chromosome 9, one of which could be considered a strong functional candidate identified by comparative annotation: *PRMD1* is a transcription repressor that acts on the beta interferon gene expression and affects the maturation of B-lymphocytes into antibody secreting cells. The *PRDM1* gene, also known as B lymphocyte induced maturation protein (*Blimp1*), has been shown to play a major role in regulating the functional differentiation of B and T lymphocytes in humans [41]–[43], and has also been implicated in myeloid function [44]. Moreover *PRDM1* interacts with several chromatin-modifying enzymes to induce transcriptional repression at the IFN-beta promoter [45]. Although this gene was not annotated in cattle, by comparative analysis we were able to locate the gene on the region of BTA9 where the significant SNP was located using the similarity with the human ortholog. Therefore *Blimp1* represents a strong candidate to be further investigated for potential association with Johne's disease. The significant SNP located on chromosome 11 in the present study falls within a QTL region found in another GWA scan for ParaTB tolerance (Settles, personal communication). This region harbours several genes: *E2F6, PQLC3* ***,*** *C2o50, KCNF1, PDIA6, ATP6V1C2, NOL10, ODC1, HPCAL1*. However, none of these genes are obvious candidates for Johne's disease susceptibility based on their known functions.

The major difficulty with studies using large numbers of tests, especially using the new high density SNP panels, is setting an appropriate correction for multiple testing. Traditional methods, such as Bonferroni correction are likely to be too strict, while relaxing the correction will increase the false discovery rate. Therefore independent replication of studies is important to confirm results. In the present study a smaller cohort of Holstein drawn from the same population, was used to test the significant associations identified in the first analysis. This second analysis confirmed 5 of the 6 moderately significant SNP associations.

Comparison of results across studies of Johne's disease in cattle identified only some regions in common. This is not surprising as the size of a study, the population structure, markers used and definition of the trait will all contribute to the significance of different genetic loci. It is therefore interesting to examine regions reported in other studies that did not pass the threshold for significance in the present study, to identify if there is any suggestion of an effect. The definition of an infected animal can be based either on serology or bacterial culture from tissue or faeces. Previous studies of heritability estimates were based on serology, while recent genome wide association study [33] used tissue and faecal culture of the bacteria as the phenotypes. Differences in the definition of infected status may explain the different genetic loci indicated as potentially involved in the susceptibility of Johne's disease. In this study MAP negative control animals were selected based on optimizing exposure to infection, considering animals that shared the same environment for the same period of time from birth. The “matched controls” are indeed not control animals in the strict sense, but can be defined as animals from the same cohort that are currently “non ELISA reactive”, compared to ELISA positive animals. The effect of misclassified animals in the “control” set would have been a loss of power of the experimental design, nonetheless, evidence of strong association with particular SNPs was identified. Using serology data will identify those loci involved in the immune response to disease, while the culture of bacteria from tissue may identify genes involved in persistence of infection at different stages of the disease. Pinedo et al [27] tested three candidate genes related to the immune function for an effect on disease*: BoIFNG* (BTA5), *TLR4* (BTA8), *SLC11A1/NRAMP1* (BTA2). In the present study a SNP on BTA8 was close to significance, however it is located more than 70 Mb from the *TLR4*.

Markers associated with resistance or susceptibility could be used in breeding strategies to reduce disease incidence. Such markers could also be the starting point in identifying the genes and hence the biological pathways, associated with response to infection with MAP which may be useful in developing diagnostic tests, or therapeutic approached to control the disease. Confirmation of the data presented here will be sought in further replication studies in independent populations as well as studies in different breeds.

In summary, the BTA9 association with antibody response to MAP found in the present study was also seen in a genome-wide association study for MAP bacterial burden [33]. Significant SNP on BTA 12 fall within a region identified in an early MAP study [32]. These two regions merit further investigation, and in particular the *PRDM1* gene on the chromosome 9 is a good positional and functional candidate.

## Materials and Methods

### 1. Animals

Samples were collected from routine Johne's disease screening of Holstein cattle carried out between September 2007 and December 2008 in the province of Lodi in Italy, in area with high occurrence of Johne's disease. All samples used in the study originated from animals routinely tested for MAP and were obtained in an anonymous form from the State Veterinary Laboratory within the framework of a local ParaTB eradication program carried out in collaboration between the breeders associations and state services. Samples were from animals belonging to infected herds with high a occurrence of Johne's disease, based on the serum antibodies produced in response to MAP infection using the ID-screen® test (Id.Vet Montpellier, France). In order to minimize relatedness between animals, as the presence of closely related individuals would confound the association analysis, samples were selected from many herds. In total 2818 samples from Holstein cows were collected from 119 farms, among which, 966 samples were chosen for the study. Of these samples 483 were MAP antibody positive (cases) and 483 MAP antibody negative (MAP negative controls). All animals were female, and cases and MAP negative controls were from the same farm tested on the same day. The ID-screen® test was used to measure serum antibodies produced in response to *M. paratuberculosis* infection (Id.Vet Montpellier, France). Cases were defined as animals serologically positive for MAP by ELISA with a sample-to-positive ratio (S/P) >0.7 and MAP negative controls were defined as animals showing a sample-to-positive ratio (S/P) <0.6 as suggested by the supplier. Furthermore, positive animals were tested twice to confirm the positive result. The Enzyme-linked immunoassay (ELISA) test is best used as a herd screening test for *M. paratuberculosis* and returns a positive result in animals that progress from an incubation phase to the clinical manifestation of Johne's disease. The ELISA test is widely used in heard health programmes to control the disease.

A further set of samples were selected to confirm results, these were: 277 Holstein cows (140 cases and 137 controls, herd, sex and test day matched). Clotted blood samples were obtained following the recovery of serum for MAP ELISA testing. DNA was extracted from the clotted blood as follows: the clot was washed with the EL buffer (NH4Cl 0.15M, KHCO3 10 mM, EDTA 0.5) and then the samples were incubated with KL buffer (SDS 1%, TRIS 1M, EDTA 0.5M, NaCl 5M) and proteinase K at 60°C for three hours. DNA was then extracted using a standard phenol-chloroform protocol, followed by an ethanol precipitation.

The 966 samples, plus 9 duplicated samples as technical replicates, were genotyped by GeneSeek Inc (Nebraska, USA) using the Illumina BovineSNP50 BeadChip which contains 54001 SNPs with an average spacing of 51.5 kb and a median spacing of 37.3 kb, based on the BTAU4.0 assembly (ftp://ftp.hgsc.bcm.tmc.edu/pub/data/Btaurus/). Genotypes were assigned using BEADSTUDIO (Illumina, San Diego) software.

### 2. Genotype quality assurance and internal population structure analysis

Genotype quality assurance was performed within the R statistical environment using the GenABEL package as implemented with the “check.marker” function [46]. Data was quality controlled for marker call rate, minor allele frequency and Hardy Weinburg Equilibrium (HWE): markers missing 5% of data, or with MAF of less than 2% were removed as were markers that were significantly out of HWE. Genotyping efficiency for samples was also verified and samples with more than 5% missing data were removed. The duplicated samples showed 99.9% concordance of genotypes calls.

Classical Multi Dimension Scaling (MDS) was used to explore population substructure and to verify the genetic homogeneity of the sample set prior to analysis. Pair wise identities by state (IBS) were calculated for all 966 samples based on autosomal SNPs using identity matrices as implemented in the GenABEL library [46].

### 3. Statistical analysis

Genome-wide association analysis was performed using the GenABEL package [46] in R using a three step GRAMMAR-CG approach, (Genome wide Association using Mixed Model and Regression - Genomic Control), with the extension of using the genomic kinship matrix estimated through genomic marker data, instead of the pedigree [39], [47]. First an additive polygenic model was used to obtain individual environmental residuals using the polygenic function of the GenABEL library to disentangle the cryptic population structure caused by the presence of closely related animals in the sample set [39]. To account for relatedness, the variance/covariance matrix was estimated from the genomic kinship matrix, as pedigree information was not available. The relationship matrix used in the analysis was estimated using genomic data with the “ibs” (option weight =  “freq”) function of GenABEL. Secondly, association was tested using a simple least squares method on the residuals, corrected for cryptic relatedness, familiar correlation, and independent of pedigree structure. Thirdly, the Genomic Control (GC) approach was used to correct for conservativeness of the GRAMMAR test, based on the estimation of the lambda factor, which is the median of all genome-wide observed test statistics divided by the expected median of the test statistic under the null hypothesis of no association, assuming that the number of true associations is very small compared to the number of tests that are actually performed.

Cases were defined as animals serologically positive for MAP by ELISA with a sample-to-positive ratio (S/P) >0.7 and MAP negative controls were defined as animals showing a sample-to-positive ratio (S/P) <0.6. Cases were set to 1 and MAP negative controls to 0. Uncorrected p-values <5×10^−7^ were accepted to represent very strong proof of genome-wide association, while p-values between 5×10^−7^ and 5×10^−5^ were considered as moderately significant associations.

SNP effects were then estimated using the formula V = 2pqa^2^ where p and q are the frequencies of the minor and major *alleles* and a is the *allelic substitution effect* [48]. Further to the initial genome-wide association study (GWAS) a confirmatory association study (CMAS) was carried out, using a smaller randomly selected sub-set of animals belonging to the same initial cohort of Holstein samples used in the initial the GWA. The analysis of these data followed the same statistical approach as described above. The threshold for confirmation of significant results in the smaller Holstein cohort was set at a p-value of less than 0.05 divided by the actual number of SNPs tested (n = 6).

SNP location and gene names were based on the Btau_4.0, assembly released on 4 October 2009 (http://www.ensembl.org). All analyses were carried out within the R statistical environment (http://www.r-project.org).

## Supporting Information

Table S1 Functional description of the genes present in the genomic regions associated with MAP within 1MB from the SNP.
(0.06 MB DOC)
