# Supplementary material for: Genetic Loci Involved in Antibody Response to Mycobacterium avium ssp. paratuberculosis in Cattle
Source: PLoS One. 2010 Jun 15;5(6):e11117. doi: 10.1371/journal.pone.0011117 (PMC2886106; doi:10.1371/journal.pone.0011117)
Supplement: Table S1 — Functional description of the genes present in the genomic regions associated with MAP within 1MB from the SNP. (0.06 MB DOC) [file pone.0011117.s001.doc]

**Supporting Information.**

**Table S1.** Functional description of the genes present in the genomic regions associated with MAP within 1MB from the SNP.

| **SNP name** | **BTA** | **Gene name (1Mb)** | **Protein name** | **Function** |
| --- | --- | --- | --- | --- |
| BTB-02056135 | 8 | [ENSBTAG00000035557](http://www.ensembl.org/Bos_taurus/Gene/Summary?db=core;g=ENSBTAG00000035557;r=8:36757076-37757076;t=ENSBTAT00000050048) |  | Pseudogene |
| ARS-BFGL-NGS-8531 | 9 | [ENSBTAT00000038553](http://www.ensembl.org/Bos_taurus/Transcript/Summary?db=core;g=ENSBTAG00000025108;r=9:45862636-46862963;t=ENSBTAT00000038553) |  | Pseudogene |
|  |  | IPI00698496.3 | *PRDM1*: **PR domain zinc finger protein 1** | **Transcriptional repressor that binds specifically to the PRDI element in the promoter of the beta-interferon gene. Drives the maturation of B-lymphocytes into Ig secreting cells.** |
|  |  | PPCE_BOVIN | *PREP*: **Prolyl endopeptidase** | Cleaves peptide bonds on the C-terminal side of prolyl residues within peptides that are up to approximately 30 amino acids long |
| ARS-BFGL-NGS-17731 | 11 | E2F6_BOVIN | *E2F* transcription factor 6 | E2F family members play a crucial role in control of the cell cycle and of the action of tumor suppressor proteins |
|  |  | NP_001095348.1 | *PQLC3*: **PQ-loop repeat-containing protein 3** | Protein coding |
|  |  | CB050_BOVIN | **Uncharacterized protein C2orf50 homolog** | Protein coding |
|  |  | NP_001091456.1 | *KCNF1*: **Potassium voltage-gated channel subfamily F member 1** | Putative voltage-gated potassium channel. |
|  |  | A6QNL5_BOVIN | ***PDIA6*: protein disulfide isomerase family A, member 6v** | Endoplasmic reticulum (ER) resident proteins that catalyze formation, reduction, and isomerization of disulfide bonds in proteins |
|  |  | NP_001077122.1 | *ATP6V****1C2*: V-type proton ATPase subunit C 2v** | Subunit of the peripheral V1 complex of vacuolar ATPase. Subunit C is necessary for the assembly of the catalytic sector of the enzyme and is likely to have a specific function in its catalytic activity. V-ATPase is responsible for acidifying a variety of intracellular compartments in eukaryotic cells. |
|  |  | NP_001069208.1 | *NOL10*: **Nucleolar protein 10** | Protein coding |
|  |  | IPI00826159.3 | *ODC1*: ornithine decarboxylase 1 | Encodes the rate-limiting enzyme of the polyamine biosynthesis pathway which catalyzes ornithine to putrescine. |
|  |  | HPCL1_BOVIN | *HPCAL1*: Hippocalcin-like protein 1 | Potentially involved in the calcium-dependent regulation of rhodopsin phosphorylation. |
|  |  | DCOR_BOVIN | *ODC1*: Ornithine decarboxylase |  |
| ARS-BFGL-NGS-57278, BTA-95991-no-rs, ARS-BFGL-NGS-101584, ARS-BFGL-NGS-105846, BTB-01470661 | 12 | NP_001075199.1 |  | Protein coding |
|  |  | IPI00827473.3 | *GPC6*: **Glypican-6** | Cell surface proteoglycan that bears heparan sulfate. Putative cell surface coreceptor for growth factors, extracellular matrix proteins, proteases and anti-proteases |
|  |  | TYRP2_BOVIN | ***TYRP2*: L-dopachrome tautomerase** | Involved in regulating eumelanin and phaeomelanin levels |
|  |  | TGDS_BOVIN | ***TGDS*: dTDP-D-glucose 4,6-dehydratase** | Protein coding |
|  |  | IPI00707374.2 | ***GPR180*: Integral membrane protein GPR180** | Protein coding |
|  |  | IPI00694817.4 | *SOX21*: **Transcription factor SOX-21** | May play a role as an activator of transcription of OPRM1 |
|  |  | IPI00701895.1 | *ABCC4*: **Multidrug resistance-associated protein 4** | May be an organic anion pump relevant to cellular detoxification |
|  |  | IPI00841680.2 | *ABCC4*: **Multidrug resistance-associated protein 4** |  |
|  |  | IPI00824465.3 | *ABCC4*: **Multidrug resistance-associated protein 4** |  |
|  |  | IPI00734394.2 | *ABCC4*: **Multidrug resistance-associated protein 4** |  |
|  |  | IPI00731343.3 | *ABCC4*: **Multidrug resistance-associated protein 4** |  |
|  |  | IPI00905426.1 |  | Protein coding |
|  |  | NM_001098469.1 |  | Protein coding |
| ARS-BFGL-NGS-37647 | 27 | IPI00843548.2 |  | Protein coding |
|  |  | IPI00843531.2 |  | Protein coding |
